# The Influence of Starch Origin on the Properties of Starch Films: Packaging Performance

**DOI:** 10.3390/ma14051146

**Published:** 2021-02-28

**Authors:** Zuzanna Żołek-Tryznowska, Alicja Kałuża

**Affiliations:** Division of Printing Technologies, Mechanics and Printing Institute, Faculty of Production Engineering, Warsaw University of Technology, Konwiktorska 2, 00-217 Warsaw, Poland; alicja.kaluza.stud@pw.edu.pl

**Keywords:** starch films, biodegradable packaging, surface free energy, starch origin

## Abstract

Starch films can be used as materials for food packaging purposes. The goal of this study is to compare how the starch origin influence the selected starch film properties. The films were made from various starches such as that from maize, potato, oat, rice, and tapioca using 50%_w_ of glycerine as a plasticizer. The obtained starch-based films were made using the well-known casting method from a starch solution in water. The properties of the films that were evaluated were tensile strength, water vapour transition rate, moisture content, wettability, and their surface free energy. Surface free energy (SFE) and its polar and dispersive components were calculated using the Owens-Wendt-Rabel-Kaelbe approach. The values of SFE in the range of 51.64 to 70.81 mJ∙m^−2^ for the oat starch-based film and the maize starch-based film. The films revealed worse mechanical properties than those of conventional plastics for packaging purposes. The results indicated that the poorest tensile strength was exhibited by the starch-based films made from oat (0.36 MPa) and tapioca (0.78 MPa) and the greatest tensile strength (1.49 MPa) from potato.

## 1. Introduction

Food packaging materials must fulfil certain criteria and perform several functions simultaneously [1]. The primary function of food packaging is to contain the products and to protect them from external factors. The packaging should contain some necessary information about the product like usage directions, nutritional details or dosage information and promote the product to attract the consumer. 

The food packaging industry is interested in developing packaging using materials that are less toxic to humans and the environment, and therefore it is constantly changing and growing. It is possible to observe this change in the resources and materials used in this industry. Unfortunately, not all of them are environmentally friendly. Some of them may cause serious damage to the environment. The most commonly used material for wrapping food in plastic, which is made from non-renewable petroleum and is non-biodegradable. They exhibit many drawbacks. They are widely used in food packaging because they ensure appropriate mechanical, chemical, and microbial preservation from external factors [2] and, at the same time good printability allows the product to be made more attractive for the consumer. The most significant advantages of plastic films are their thermosealability, wide range of thermal and mechanical properties, lightness, and low price. Plastic films permit integrated processes, that is, plastic packages can be formed, filled, and sealed continuously within a production line. Furthermore, the storage of plastics does not require special conditions in comparison to, for example, paper or cardboard materials. In the food packaging industry, the most frequently used polymers are polyethylene (PE), polypropylene (PP), polystyrene (PS), polyvinyl chloride (PVC), and polyethylene terephthalate (PET) [2,3]. These plastics are obtained from petroleum raw materials, and they have become a significant source of waste [2]. Flexible films used primarily as packaging are usually simply sent to a landfill without considering the possibility of recycling. Hence, to protect human health and the environment, the principal waste reduction of plastic food packaging should be considered. Plastic materials have already polluted roads, forests, mountains, and our oceans [4]. The problem is not the non-degradability or the very long time it takes plastics to break down in the natural environment, but also the presence of microplastics [5] (<5 mm in size) in our oceans. Another major disadvantage of plastics is the emission of toxic gases formed during the production of various fillers and special colours used to improve the plastic’s properties, depending on customer requirements [6].

Thus, one of the fundamental challenges is to reduce the effect of human and industrial activities on the natural environment. Nowadays, efforts are being made to find alternatives and ecological substitutes for currently known polymers [7] and plastic packaging, and as a solution starch films may be used [8]. Starch is a biopolymer, degradable by microorganisms such as fungi, bacteria, and enzymes [9]. Starch is formed in nature and can be used as a raw material for biodegradable materials [10,11,12,13,14,15,16,17]. Furthermore, starch-based films might be a less expensive choice than polylactic acid (PLA) films for some food packaging. Since the 1980s, the filmogenic properties of starch materials have been a subject of intense interest [18]. Unlike other biopolymers, starch is abundant, cheap, biodegradable, and edible, with an excellent filmogenic capability [3,19,20,21,22,23,24,25]. Hence, starch seems to be one of the most promising choices to replace petroleum-based plastics. Starch-based films and composites offer great potential to be ecologically suitable materials for food packaging [3,20,22,23,26]. Starch-based film may be used to create packaging that has antibacterial and antioxidant properties [17,27,28,29,30,31]. Starch has application in various fields—primarily in the food, pharmaceutical, cosmetic, and paper industries—as a matrix, binder or filler [32,33]. Some unfavourable properties of natural starch, like insolubility in cold water, prevent its wider use. However, starch processing, such as modification by chemical, enzymatic and physical methods, can change these properties, thereby making them attract a lot of interest from potential buyers [32]. This processing must be done by the processing unit’s specialized operations, such as ultrasound treatment [32,34,35,36]. 

The primary sources of starch are agricultural plants (e.g., potato, rice, corn). Starch is a polysaccharide made of glucose units bounded by α-glycosidic bonds. In plants, the starch is formed as hydrophilic granules composed of different types of polysaccharides: a linear and crystalline amylose (poly-α-1,4-d-glucopyranoside), branched and amorphous amylopectin (poly-α-1,4-d-glucopyranoside), and α-1,6-d-glucopyranoside) [37]. Maize, wheat, potato, and rice starches take the world market leaders: 84, 7, 4, and 1%, respectively [38]. The original starch chemical composition is based on two macromolecular components: amylose and amylopectin [39]. However, starches have varied structures that are influenced by amylose and amylopectin content and the dimension of starch granules.

This comparative work–study of the selected properties of starch films derived from various food sources (maize, potato, oat, rice, and tapioca) was performed. Starch films were obtained with 50%_w_ addition of glycerol as a plasticizer. This amount of plasticizer allows the production of films with good mechanical properties. For packaging purposes, the material must have a suitable surface, barrier, and mechanical properties to maintain the product’s safety. Therefore, the tensile strength, water vapour transition rate, and surface free energy of the obtained starch films were evaluated. All the samples were prepared, and measurements were conducted under the same conditions. To the best of our knowledge, similar comparisons have not yet been reported in the literature. 

## 2. Materials and Methods

### 2.1. Materials

Maize (NaturAvena, Piaseczno, Poland), potato (Bio Planet, Leszno, Poland), oat (Bio Planet, Leszno, Poland), rice (Sigma-Aldrich, Poznań, Poland), and tapioca (Bio Planet, Leszno, Poland) food starches were used. Glycerol (purity ≥99%, CAS 56-81-5) was used to maintain the proper flexibility of films. The liquids used for contact angle measurements were diiodomethane (purity ≥99%, CAS 75-11-6, Sigma–Aldrich, Poznań, Poland), formamide (purity ≥99%, CAS 75-12-7, Sigma-Aldrich), and water. Water was purified by electrodeionization with MilliporeSigma Elix Water Purification System (Burlington, MA, USA). All chemical reagents were used as received. 

### 2.2. Preparation of Starch Solution

Starch (10 g) and 5 g of glycerol were added to 200 g of water. Next, the starch solution was heated (3 °C/min) and continuously mechanically stirred with Heidolph RZR 2020 (Schwabach, Germany) set at 500 rpm. The solution was heated to 95 °C, which was guaranteed to exceed the used starch’s gelatinization temperature (see Table 1). Next, the starch solution was held for 5 min before cooling to 50 °C.

### 2.3. Film Formation

The starch solution was cast on a Teflon plate. K Paint Applicator (RK Prints, Royston, UK), equipped with an adjustable micrometer 3 mm spreader gap with a constant coating speed (6 m∙min^−1^), was used to obtain repeatable films. Then 200 g of the starch solution was allowed to receive eight sheets of starch film with dimensions of ~100 mm × 150 mm. The films were dried in a climate chamber at room temperature (23 ± 0.5 °C) and humidity (50 ± 1% RH). The films were peeled from the Teflon plates by hand. To ensure the measurements’ repeatability, each type of film was prepared from two different starch solutions. All analyses were conducted on the film side exposed to the air and one week after the film was obtained.

### 2.4. Estimation of Packaging Properties of Starch Films

The mechanical properties, contact angle measurement, water vapour transition rate, and moisture content were performed according to the procedure described in our previous work [48].

#### 2.4.1. Mechanical Properties

The thickness of the films was measured using a digital thickness gauge FD-50 (Käfer, Villingen-Schwenningen, Germany) with a resolution of 1 µm with an error ±5 µm. Data collection was performed at eight different positions of the samples.

The tensile strength, Young’s modulus, and elongation at break were measured with a Z010 tensiometer (Zwick-Roell, Ulm, Germany). Stripes were prepared to conform to ISO 527-1. The stripes had dimensions of 100 mm × 15 mm. According to ISO 527-1 [49], the grip’s initial distance was 50 mm; the stretching speed was 100 mm min^–1^. The strain-stress curves were used to calculate Young’s modulus. For each kind of starch film, all specimens were estimated eight times from two different films.

#### 2.4.2. Surface Properties

The contact angle was measured by the sessile drop method using the drop shape analysis system DSA 30E (Krüss, Hamburg Germany). Drops of the liquids were deposited on a starch film. To test wettability, a picture of water on the film surface was taken. The Advance (Krüss, Hamburg, Germany) software was used for the drop shape analyses. The measurements were performed in a climate chamber to ensure stable conditions (23 ± 0.5 °C, 50% RH). The SFE was calculated using the Owens-Wendt-Rabel-Kaelbe [50], and the van Ossy-Chaudhury-Good [51] approaches.

#### 2.4.3. Water Vapour Transmission Rate

To test the water vapour transmission rate (WVTR), a disc with a 54 mm ± 2 mm diameter was cut out and placed in a MA 210.R (Radwag, Radom, Poland) moisture analyzer on an aluminum-sealed probe with 5 g of water and weighed. Next, the film samples were placed into the cells. The temperature was set to 45 °C. The room temperature (23 ± 0.5 °C) and relative humidity (50 ± 1% RH) were constant. The measurements were done manually, and the samples were weighed at intervals of 0, 1, and 2 h. The WVTR was calculated using Equation (1)

(1) $$WVTR=\frac{\Delta m}{t\cdot s},$$

where: Δ*m* is the difference between the mass of water after 2 and 1 h of measurement; *t* is the time of measurement (1 h), and *S* is the area of the film sample. The measurements were performed three times.

#### 2.4.4. Moisture Content

The moisture content of all film samples was measured using MA 210.R (Radwag, Radom, Poland). They were dried in a furnace at 105 °C ± 3 °C for approximately 12 h. Moisture content was calculated as a percentage mass loss during the drying of the films. The measurement was repeated in triplicate.

### 2.5. Statistical Analyses

Statistical analysis was performed by using the Statgraphics Centurion 18 (v.18.1.06 StatPoint^®^, Inc., Warrenton, VA, USA) software. One-way ANOVA was used to analyze the data. The Tukey test with *p* < 0.05 as statistical significance was used to evaluate the mean values.

## 3. Results

### 3.1. Film Appearance

**Figure 1:**
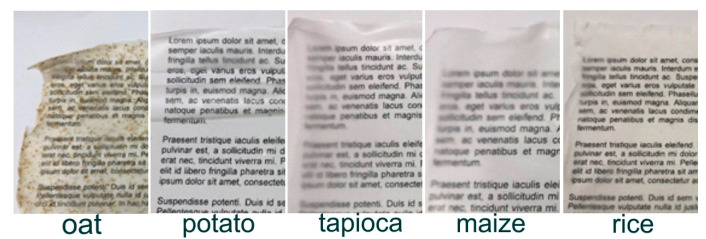
The appearance of various starch films.

The starch films’ visual appearance is summarized in Table 2, and the samples of the starch films are shown in Figure 1. The films were odourless and transparent. During storage, shrinkage of the films was observed. Although the edges of the foil were brittle, the films had an acceptable appearance, and they could be peeled from the Teflon plates without failure so that they could be used for further testing.

Appearance may be a crucial factor when using starch-based film for packaging production. Good transparency allows for film printing and legible reading of inscriptions. If reading were difficult or impossible, none of the packaging manufacturers would opt for this solution due to the loss of the product’s aesthetic value. Films made from tapioca, rice, and maize starch exhibited good transparency. However, those made from rice and maize starch showed a slightly yellowish color, which may limit their use in the packaging industry or necessitate undercoats to obtain the desired final color of the film.

The oat film exhibited the lowest transparency because of the thickening on the film’s surface, which made it difficult to read the text under the film. This film’s texture is also different—bumps can be seen with the naked eye and felt under finger pressure. The potato film also exhibited lower transparency in comparison to those made from tapioca, maize, or rice. The potato films’ wrinkling during storage may be related to the external conditions (low humidity, 50% RH) under which the films were stored. The influence of storage humidity on the properties of starch films has been previously reported [18].

### 3.2. Mechanical Properties

Table 3 presents the mechanical properties of the starch films. In general, they exhibited inferior properties to conventional films. Modern biodegradable plastics used in the packaging industry exhibited higher values of tensile strength or elongation at break (e.g., 13.5 to 35.5 MPa with 400% and 30% elongation at break for Mater-Bi^®^ (Novamont, Novara Italy) and PLA (NatureWorks, Minnetonka, MN, USA), respectively [52]. In contrast, the tensile strength samples investigated in this study varied from 0.36 ± 0.05 to 3.05 ± 0.66 MPa, with the lowest value of tensile strength being observed for the oat film and the highest value for the potato film. Because the tensile strength of starch-based films is much lower than that of modern biodegradable plastics in the packaging industry, their scope of application is limited. This may be related to the amylose and amylopectin content as well as to grain morphology.

Moreover, the highest value of elongation at break was determined for the tapioca film (137 ± 5%), while the oat film exhibited the lowest value (27 ± 5%).

In general, tensile strength is directly related to the Young’s modulus and inversely associated with elongation at break. The elasticity of the starch-based films was related to the interactions of the starch molecules influenced by the plasticizer. The Young’s modulus was in the range of 0.8 ± 0.3 MPa to 14.5 ± 10.6 MPa for the maize and potato films, respectively.

All mechanical property parameters presented a statistical difference (*p* < 0.05) among the starch films, despite the quite significant error for the Young’s modulus of maize and potato films.

### 3.3. Wettability and Surface Free Energy

The contact angle and surface free energy indicated surface hydrophobicity or hydrophilicity. Surface wettability, together with moisture content and water vapor permeability, gave an overview of the starch-based materials’ performance in everyday conditions. The static water-contact angle values provided beneficial information about the wetting of the solid by water and its wettability properties.

**Figure 2:**
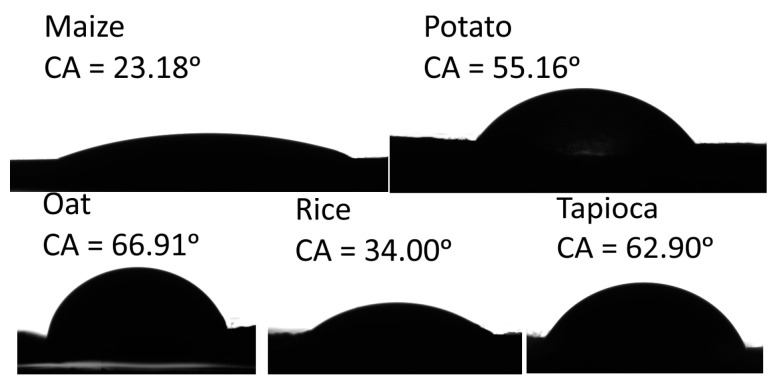
The contact angle of water droplets on starch-based films.

The values of the water contact angle (CA) and the calculated values of surface free energy are summarised in Table 4. For instance, the (CA) values indicated the hydrophilicity (CA < 90°) or hydrophobicity (CA > 90°) of the surface [53]. A more hydrophobic surface is desirable because a starch-based film’s main drawback is its affinity to water, which limits its usage. The images of water CA on the starch-based films are presented in Figure 2. The images clearly show lower hydrophilicity for the oat and tapioca films and higher hydrophilicity of the maize and rice films. The results show that the water CA values ranged from 23.18 to 66.91° for the maize and oat films, respectively. Significant differences were shown between the water CA and starch origin. We suspect that the water CA variation is due to differences in amylose and amylopectin contents and, simultaneously, the size of the starch molecules and the degree of polymerization, which is comparable for tapioca and potato and much lower for maize, as reported by Swinkels [54]. The values of water CA give useful information about its wettability. Wettability for the maize film is the highest, and the lowest is for oat film.

There are a few approaches that allow for the calculation of SFE from a measured contact angle data. However, the Owens-Wendt-Rabel-Kaelbe method, also known as the Owens-Wendt [50] method, is commonly used to measure surface free energy. Thus, in this work, the Owens-Wendt-Rabel-Kaelbe (OWRK) method was used. A detailed description of the surface free energy determination of starch-based materials can be found in [48,50].

The surface free energy and the water contact angle are crucial parameters of ink transfer on the film [55]. The values of total the SFE were much higher, in the range of 51.64 to 70.81 mJ∙m^−2^, for the oat and maize films. These values were higher than those previously measured for plastic films. The reported values of SFE for LD-PE, PP, and PLA were 35.3 mJ∙m^−2^ [56], 32.3 mJ∙m^−2^ [57], and 49.7 mJ∙m^−2^ [55], respectively. The value of the polar component of the SFE was much lower than the values of the dispersive component, regardless of the presence of polar groups, like hydroxyl groups, which are responsible for polar interaction with polar liquids. These polar groups are responsible for the increase in interfacial free energy [58]. Moreover, values of total SFE and its polar and dispersive components significantly varied among the different starches used for the films (*p* < 0.05).

**Figure 3:**
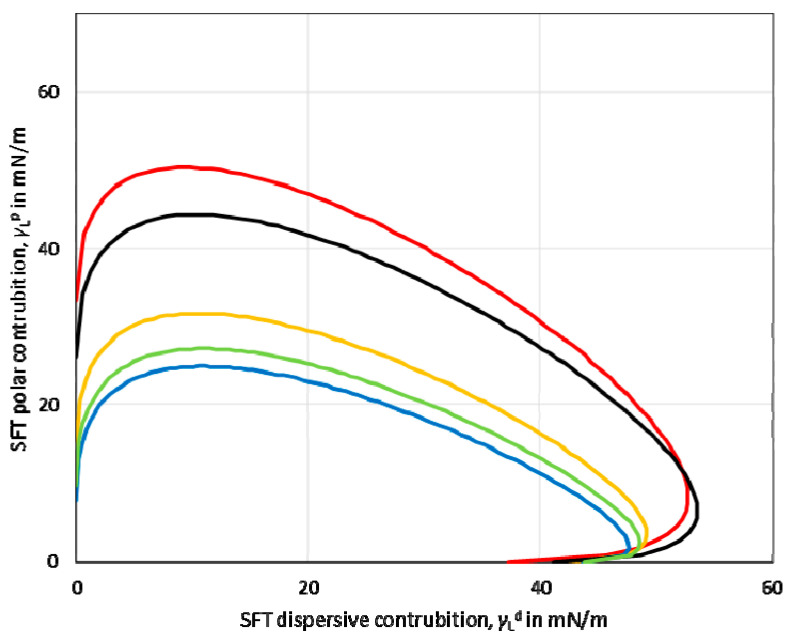
Surface energy wetting envelope calculated for maize (red line), potato (yellow line), oat (blue line), tapioca (green line), rice (black line) starch-based films.

Based on the values of the dispersive and polar components, the so-called wetting envelope of a polymeric film’s surface properties was calculated. The wetting envelope is the fastest method that predicts the wetting behaviour of the liquids on the substrates. A wetting envelope is plotted when the polar and dispersive parts of the substrate’s surface free energy are known. Figure 3 shows the wetting envelopes for the obtained starch-based films. It can be seen that the wetting envelopes of the films made from potato, tapioca, and oat starch were comparable. Furthermore, the wetting envelope of the maize starch-based film (red line) exhibited a larger area than the other investigated starches.

### 3.4. Water Vapour Transmission Rate, Moisture Content

The WVTR and moisture content are listed in Table 5. The WVTR indicates the amount of water vapor that can permeate the packaging material’s unit area per unit of time [59]. The WVTR gives an overview of the barrier properties of packaging. The measured values of the WVTR are similar to values previously reported for starch films. For example, Xu et al. has reported the values of WVTR for chitosan-starch films in the range of 4.64 to 5.27 mg∙cm^−2^∙h^−1^ [60]. For the WTVR, no statistical difference (*p* > 0.05) was observed between the starch types.

The lowest moisture content was observed for the potato film (9.74 ± 2.22%), which was observed to manifest film breakage and low flexibility. The highest moisture content was determined for the maize film, equal to 22.26 ± 2.33%. It should be highlighted that the drying procedure and environmental conditions during drying were kept constant (23 °C, 50% RH, seven days). The moisture content of the films varied significantly among the different starches (*p* < 0.05).

## 4. Conclusions

To analyse the influence of starch origin on the film properties, the films’ basic properties such as mechanical properties, wettability and surface free energy, water vapour transition rate, and moisture content were analysed.

The starch-based films were prepared using five different food starches––maize, potato, oat, rice, and tapioca––with the addition of 50%_w_ of glycerine as a plasticizer. Our study revealed that starch-based films made from oat and tapioca exhibited the lowest tensile strength (0.36 and 0.78 MPa, respectively) On the other hand, the highest values of tensile strength were observed for films made from potato starch (3.05 MPa); however, these values are 10 times lower than those for modern biodegradable packaging films. The tensile properties of starch-based films strongly limit their use for packaging purposes.

Furthermore, the oat and tapioca films exhibited the highest contact angle values and, simultaneously, the lowest values for the polar component of the surface free energy, which is related to lower wettability and lower hydrophilicity. All the investigated starch-based films exhibited high surface free energy.

Our results expand the knowledge of using starch-based films as packaging materials. Despite their disadvantages, they might be feasible for single-use packaging. Basic functional properties such as mechanical properties, surface free energy, and other starched-based film parameters show their possible use for packaging purposes. Research should be focused on improving the packaging properties of starch-based films.

## Figures and Tables

**Table 1:** Gelatinization temperature and amylose content of various starches.

Starch Type	Temperature (°C)	Amylose (%)
Maize	64.4 [40]	22.5 [40] to 29.4 [41]
Potato	58.2 [40]	21 [42] to 26 [43]
Oat	60.4 [42]	17.3 [44] to 33.6 [45]
Rice	71.1 [40]	20 [46]
Tapioca	62.4 [40]	17 [43] to 20 [47]

**Table 2:** External features of various starch films.

Type of Starch Film	Transparency	Smell	Flexibility	Extensibility
Maize	+	+	+	+
Potato	+	+	−	−
Oat	+/−	+	+	+
Rice	+	+	+	+
Tapioca	+	+	+	+

+ observation; − no observation.

**Table 3:** Properties of obtained films.

Film	Tensile Strength (MPa)	Elongation at Break (%)	Young’s Modulus (MPa)	Thickness (µm)
Maize	1.49 ± 0.26 ^b^	51 ± 6 ^b^	14.2 ± 6.7 ^c^	266.8 ± 0.4 ^b^
Potato	3.05 ± 0.66 ^c^	70 ± 8 ^b^	14.5 ± 10.6 ^c^	332.7 ± 4.4 ^b^
Oat	0.36 ± 0.05 ^a^	27 ± 5 ^a^	1.8 ± 0.9 ^ab^	266.9 ± 1.0 ^c^
Rice	1.80 ± 0.39 ^b^	49 ± 3 ^ab^	9.6 ± 0.3 ^bc^	145.1 ± 2.5 ^a^
Tapioca	0.78 ± 0.22 ^a^	137 ± 5 ^c^	0.8 ± 0.3 ^a^	136.5 ± 5.9 ^a^

Values are means (n = 8) ± SD. Means in the same column with the same letter are not significantly different (*p* < 0.05).

**Table 4:** The values of water contact angle and surface free energy components.

Film	Contact Angle ^1^ (°)	Surface Free Energy (mJ∙m^−2^)		
	Water	Dispersive	Polar	Total
Maize	23.18 ± 2.19 ^a^	37.34 ± 0.59 ^a^	33.47 ± 1.01 ^d^	70.81 ± 0.85 ^b^
Potato	55.16 ± 4.76 ^c^	42.40 ± 2.29 ^ab^	14.09 ± 2.26 ^b^	56.49 ± 3.64 ^a^
Oat	66.91 ± 6.08 ^d^	43.79 ± 5.50 ^b^	7.86 ± 2.84 ^a^	51.64 ± 5.43 ^a^
Rice	34.00 ± 7.08 ^b^	41.26 ± 2.25 ^ab^	26.13 ± 4.14 ^c^	67.39 ± 3.42 ^b^
Tapioca	62.90 ± 7.68 ^cd^	43.89 ± 2.37 ^b^	9.68 ± 3.74 ^ab^	53.57 ± 4.66 ^a^

^1^ measured after sessile drop deposition at the first contact. Values are means (n = 6) ± SD. Means in the same column with the same letter are not significantly different (*p* < 0.05).

**Table 5:** Water vapour transmission rate (WVTR) and moisture content of various starch films.

Type of Starch Film	WVTR (mg/(cm^2^∙h) *	Moisture Content (%) **
Maize	4.66 ± 0.02 ^a^	22.26 ± 2.33 ^b^
Potato	4.60 ± 0.04 ^a^	9.74 ± 2.22 ^a^
Oat	4.65 ± 0.02 ^a^	21.77 ± 1.48 ^b^
Rice	4.64 ± 0.02 ^a^	18.72 ± 4.67 ^b^
Tapioca	4.67 ± 0.05 ^a^	17.22 ± 1.46 ^b^

Values are means (* n = 4 or ** n = 3) ± SD. Means in the same column with the same letter are not significantly different (*p* < 0.05).

## Data Availability

The data presented in this study are openly available in Mendeley Data at doi:10.17632/m8wn5y2wny.1.
